# Compilation and Application of the Scale of Sustainable Knowledge Sharing Willingness in Virtual Academic Community During the Times of the Coronavirus Pandemic (COVID-19)

**DOI:** 10.3389/fpsyg.2021.627833

**Published:** 2021-07-15

**Authors:** Huaruo Chen, Fei Liu, Ya Wen, Ling Ling, Xueying Gu

**Affiliations:** ^1^School of Education Science, Nanjing Normal University, Nanjing, China; ^2^Center for Research and Reform in Education, Johns Hopkins University, Baltimore, MA, United States; ^3^School of Education Science, Huaiyin Normal University, Huaian, China; ^4^School of Teacher Education, Nanjing Xiaozhuang University, Nanjing, China; ^5^Institute of Mathematics and Physics, Beijing Union University, Beijing, China

**Keywords:** Heuristic-System Model, academic virtual community, COVID-19, sustainable knowledge sharing, willingness

## Abstract

With the outbreak of COVID-19, many offline academic activities have been turned online, and virtual academic communities have been further emphasized. Based on this situation, this study took the Eagly and Chaiken’s Heuristic-System Model of Persuasion and the general rules of behavioral decision as a theoretical basis, established a theoretical model of sustainable knowledge sharing willingness in virtual academic communities. Firstly, this study developed the scale of willingness to share sustainable knowledge based on the heuristic system model of persuasion. After analyzing the data of 62 participants, the scale was revised. Secondly, 256 valid data were collected from China, the United States, Singapore, and Indonesia. Finally, the conceptual model and theoretical hypothesis were tested based on the data. The results show that knowledge sharing satisfaction is affected by heuristic factors (knowledge sharing quantity, knowledge source credibility) and system factors (knowledge sharing quality, knowledge sharing usefulness), and has a significant positive correlation with sustainable knowledge sharing willingness.

## Introduction

With the rapid development of modern information technology and the popularization and application of the Internet, new digital scientific research environments such as E-Learning are increasingly formed and mature. Especially under the influence of current COVID-19, new knowledge sharing mode with an virtual academic community as the carrier has emerged and received extensive attention (Castaneda and Cuellar, 2020). The virtual academic community will gather researchers with common or similar research interests together to release questions, discuss questions, provide answers and share knowledge around the same topic, to realize knowledge sharing (Marquez et al., 2016). The virtual academic community has broken through the time, space, and discipline restrictions changed the knowledge production mode based on disciplines and documents, complied with the needs of real-time scientific research, interactive scientific research, open scientific research, and collaborative scientific research in the network era, which become an important platform for researchers to share knowledge (Cantor, 2019).

In recent years, with the rapid development of virtual academic community, the scale of community users such as Academia, ResearchGATE, Mendeley, etc. has been expanding, which not only allows users to share and view the latest scientific research results in time but also helps users to establish community relations, to make academic exchanges more smooth and knowledge sharing more efficient. But at the same time, there are also some academic virtual communities with low user participation, less knowledge sharing activities, and users’ sustainable use intention declining. The establishment soon began to decline, which did not achieve the purpose of academic exchange. From the perspective of knowledge management, virtual academic community constructs a new paradigm of knowledge production, storage, sharing, and utilization, which provides the resources, technology, and environment needed for knowledge sharing, while knowledge sharing provides power and guarantee for the sustainable development of the virtual academic community (Cheng et al., 2018). Therefore, the sustainable participation of users in knowledge sharing is the key to the success of the virtual academic community (Chandran and Alammari, 2020). In this paper, the Heuristic-Systematic Model of Persuasion (HSMPP) (Chaiken et al., 1989) and the general rules of human behavior decision-making are used to construct a heuristic for sustainable knowledge sharing in virtual academic community and the scale was formed. The systematic model explores the influence of heuristic variables and systematic variables on the willingness of sustainable knowledge sharing and analyzes the obstacles and countermeasures of sustainable knowledge sharing in virtual academic community.

## Literature Review

### Sustainable Knowledge Sharing in Virtual Academic Community

The sustainable development of virtual academic community depends on whether users are willing to share knowledge sustainably. The main challenge is that knowledge in the virtual academic community is non-competitive and non-exclusive, which is usually regarded as “public goods” (Rice et al., 2018). Cabrera and Cabrera (2002) believed that all people in an organization, whether they have contributed knowledge or not, can obtain shared resources, and the use of knowledge resources by each person will not reduce the use of these resources by others (Cabrera and Cabrera, 2002). The public goods attribute of knowledge tends to lead to the imbalance of sharing. People are always willing to obtain and use free knowledge resources, rather than contribute their own knowledge, which leads to “free-riding” behavior. However, according to Simon’s “limited rationality” theory, everyone is limited rationality, and community members may share knowledge because of irrational factors such as interpersonal relationships and emotion (Cristofaro, 2017). Therefore, there are many factors influencing knowledge sharing and sustainable willingness.

At present, the research mainly focuses on the endogenous factors such as emotional factors, psychological cognition, individual motivation, or the external variables such as technical function, social impact, situational environment to study the sustainable willingness in knowledge sharing of virtual academic community. Ranjbarfard and Sureshjani (2018) constructed a framework for knowledge sharing in virtual academic community between teachers and students, and studied the role of partnership requirements, collaborative learning services, and social networks on the willingness to sustainable knowledge sharing (Ranjbarfard and Sureshjani, 2018). Cheung and Lee (2007) pointed out that internal motivation has a strong correlation with knowledge self-efficacy, which has a significant positive impact on the willingness to continue knowledge sharing (Cheung and Lee, 2007). Chiu et al. (2011) constructed a model based on expectation recognition theory and fairness theory, pointed out that the uncertainty of self-worth, fairness of distribution, and fairness of interaction significantly affect the satisfaction and willingness of members of virtual community (Chiu et al., 2011). The existing researches have made fruitful results by using the inherent model of classical theory, but they have not distinguished the heuristic behavior and systematic behavior of knowledge sharing, and have not yet studied the rational and irrational factors and their mechanism of knowledge sharing behavior.

### Application of HSMP

The HSMP is a dual processing theoretical model proposed by psychologist Chaiken to explain the process of individual information behavior (Chaiken et al., 1989). Chaiken believed that human social activities have two kinds of information processing modes: heuristic and systematic (Chaiken et al., 1989). The heuristic behavior based on intuition means that people pay less cognitive effort and make a simple judgment according to the external clues of information (Chaiken, 1980). For example, the implication of source credibility may trigger the rule that trust means right, making people more willing to accept information sent by people with high trust. Systematic behavior based on rationality means that people use enough cognitive resources to systematically evaluate relevant information content (Chaiken, 1980). Users’ evaluation of information quality mainly considers the information content itself (such as discussion quality and discussion intensity), not only the non-content factors such as information source reliability and information quantity.

HSMP provides an in-depth theoretical explanation for how individuals deal with information, evaluate information, use information, and form decision-making in different situations, which is widely used to explore the influencing factors and situational conditions of heuristic and systematic information behavior (Chandran and Alammari, 2020). Wirth et al. (2007) proposed that information search behavior can be divided into heuristic and systematic patterns, and the importance of search experience and search results is the main factor to distinguish the two behavior patterns (Wirth et al., 2007). Lucassen et al. (2011) pointed out that for Wikipedia, students with a high degree of trust tend to adopt heuristic information behavior mode, and pay more attention to the quantity of information. On the contrary, they tend to adopt systematic information behavior mode and pay more attention to the quality of information (Lucassen et al., 2011). Zhang et al. (2014) believed that consumers’ acceptance of online comment information is a dual process, including heuristic and systematic behaviors, information source reliability and comment quantity cognition are heuristic variables, comment quality is systematic variables, and both variables have a significant impact on consumers’ behavior attitude (Zhang et al., 2014).

At present, there are little researches on knowledge sharing using HSMP. Compared with the technology acceptance model and user satisfaction model, the advantages of HSMP lie in that the model is not a fixed theoretical model composed of several specific variables, but a general framework and behavior paradigm of behavior decision-making research, which has a strong theoretical expansion and explanatory power. Using HSMP to study the sustainable knowledge sharing of virtual academic community can identify the key influencing factors and mechanism of knowledge sharing satisfaction and sustainable willingness from the general rule of behavior decision-making, without the limitation of intrinsic variables and their relations.

## Research Model and Hypothesis

### Hypotheses in Satisfaction Model

Satisfaction refers to the recognition degree of users for products, services, and behavior processes, including the evaluation after adoption and the feeling state formed in the use process (Changchit and Klaus, 2020). Satisfaction has a stable positive correlation with the user’s intention to continue to use, which can predict the user’s intention to continue to use (Bae, 2017). For the virtual academic community, user satisfaction is the premise of its sustainable development. If the user is not satisfied, it will reduce community activities and even cancel the account (Borcsa and Pomini, 2017). Therefore, in the virtual academic community, the satisfaction of knowledge sharing has a positive impact on sustainable willingness, and the relationship between them is as follows:

• H1: there is a positive correlation between satisfaction of knowledge sharing and sustainable knowledge sharing willingness in virtual academic community.

### Relevant Hypotheses of HSMP

Knowledge sharing behavior in virtual academic community is a complex dual process and has two kinds of behaviors: heuristic and systematic, which are affected, respectively. The direct measurement of heuristic and systematic cues is to see the amount of information processed and the degree of fine processing, which is difficult to operate. Some scholars try to use indirect measurement to explore two kinds of clues, that is, to investigate people’s processing methods of information content characteristics and external characteristics. Zhang et al. (2014) regarded the quantity perception and source credibility of online reviews as clues of heuristic behavior, and the cognition and discussion intensity of information degree as clues of systematic behavior to study the impact of online reviews on online shopping (Zhang et al., 2014). The behavior pattern regards the top of the page as a heuristic behavior and the middle position as the system behavior according to the location where the user clicks on the search page (Kim et al., 2014; Ghoses et al., 2018).

Because the HSMP does not put forward specific criteria for dividing heuristic and systematic behaviors, the academic community has not formed a unified view on the measurement scale of the two behaviors (Schemer et al., 2008). According to the research of Chaiken (1980), explicit factors such as external cues of behavior and formal characteristics of information are regarded as heuristic variables, potential factors such as central cues of behavior and internal characteristics of information are regarded as systematic variables, and reliability and quality are the most important influencing factors of initiating and systematic behaviors, respectively (Chaiken, 1980). In the process of knowledge sharing in virtual academic community, knowledge quality and usefulness judgment need more cognitive resources to analyze the content and value of knowledge sharing, so it can be used as the influencing factor of systematic behavior. The judgment of knowledge quantity and credibility is relatively simple thinking of external available clues, which consumes relatively less cognitive resources, and can be used as an influencing factor of heuristic behavior.

#### Relevant Hypotheses of Systematic Variables

The quality and usefulness of knowledge sharing are two main indicators to measure the level of knowledge sharing, which reflect the value of knowledge sharing among members of virtual academic community. Many studies show that quality, usefulness, and satisfaction of knowledge sharing are related. Bouncken and Aslam (2019) pointed out that the higher the quality of shared knowledge, the more expected it is, the higher the user satisfaction (Bouncken and Aslam, 2019). Gang and Ravichandran (2015) pointed out that usefulness is an important factor affecting community satisfaction (Gang and Ravichandran, 2015). Therefore, if the virtual academic community can provide users with timely and highly relevant knowledge to discuss topics, and increase users’ useful awareness of knowledge sharing, then users’ satisfaction with the knowledge sharing process will be improved. Based on this, this paper proposes the following assumptions:

• H2: there is a positive correlation between quality and satisfaction of knowledge sharing in virtual academic community.
• H3: there is a positive correlation between the usefulness and satisfaction of knowledge sharing in virtual academic community.

#### Related Hypotheses of Heuristic Variables

The credibility of knowledge source refers to the users’ overall perception of the credibility of knowledge source, including the reliability and professionalism of knowledge source, in which the reliability is related to the familiarity of community members to knowledge contributors and the recognition of knowledge. Professionalism is related to the professional experience, academic influence, and social identity of knowledge contributors in relevant fields. When people adopt heuristic behavior, they usually regard source credibility as the main basis for decision-making and judgment, and think that “expert opinion is correct” and “expert means authority and reliability” (Bonner et al., 2006). Boratto et al. (2016) showed that persuasive information with high source reliability can stimulate users’ positive evaluation (Boratto et al., 2016). Therefore, this paper holds that there is the following relationship between the credibility of knowledge source and the satisfaction of knowledge sharing in virtual academic community:

• H4: there is a positive correlation between the credibility of knowledge source and satisfaction of virtual academic community.

Quantity of knowledge sharing is another important heuristic clue, which plays an important role in user satisfaction evaluation (Altman et al., 2018). This paper studies the numbers of knowledge sharing from four aspects: total knowledge, the information contained, update frequency, and several participants. When people take heuristic evaluation to the satisfaction of knowledge sharing, they often judge the significant characteristics and external performance of knowledge sharing simply according to experience and intuition. Many studies also use quantity as a heuristic variable. Chaiken (1980) took the amount of information and the preferences of information recipients as the influencing factors of the evaluation of the information reception effect (Chaiken, 1980). Gao et al. (2012) found that the more the amount of reference information, the more conducive to reducing the differences in users’ expectations of products and improving users’ satisfaction (Gao et al., 2012). Accordingly, the following assumptions are proposed:

• H5: there is a positive correlation between the quantity of knowledge sharing and satisfaction of virtual academic community.

### Hypotheses Between Heuristic Variables and Systematic Variables

According to the HSMP, heuristic behavior and systematic behavior can occur at the same time, and the two behaviors interact with each other, resulting in a certain deviation in the final behavior. Specifically, if the two behavior results are similar, user behavior has the characteristics of both heuristic and systematic behavior patterns. The behavior results are intuitive and rational, and the two behaviors have an additive effect. If the results of the two behaviors are different, they need to further investigate the specific situation. If the situation information is clear and the conditions are clear, then the systematic behavior has a weakening effect on the heuristic behavior. People tend to adopt the system behavior based on rational judgment, otherwise, the heuristic behavior is dominant, people tend to adopt the heuristic behavior based on intuitive judgment, and produce irrational deviation.

In the process of satisfaction evaluation and decision-making of knowledge sharing in virtual academic community, the credibility of knowledge source and quantity of knowledge sharing can stimulate users’ cognition of the usefulness of knowledge and actively infer the sharing results. Chinn and Rinehart pointed out that the credibility of knowledge sources has an important impact on perceived usefulness (Chinn and Rinehart, 2016). When it is difficult for community members to judge the value of subject knowledge, if the credibility of these knowledge sources is high, and the amount of knowledge shared is large, then the members are likely to think that knowledge is of high usefulness. Therefore, this paper proposes the following assumptions:

• H6: there is a positive correlation between the credibility of knowledge source and perceived usefulness in virtual academic community.
• H7: there is a positive correlation between the quantity of knowledge sharing and perceived usefulness in virtual academic community.

### Relevant Hypotheses of Social Impact Variables

Virtual academic community is a social organization based on a network. Knowledge sharing among community members is a social exchange activity. Its process and results are affected by social capital factors. According to the theory of social exchange, people follow the principle of interest exchange in the process of knowledge sharing, exchange other people’s knowledge by contributing knowledge or expect similar help in the future, to achieve mutual benefit (Park et al., 2015). The expectation based on mutual benefit represents the invisible norm of “mutual debt,” which can be understood as a strong sense of fairness coexisting in giving and acquiring. Only when knowledge contribution is rewarded, can community members effectively stimulate their willingness to continuously contribute their knowledge? Ganguly et al. (2019) showed that reciprocity has an important impact on the quality and quantity of knowledge sharing, and knowledge-collectors must return equal or more knowledge to their contributors to maintain knowledge exchange activities (Ganguly et al., 2019). As an important relational social capital, reciprocity can help people realize the potential value of knowledge sharing, and promote knowledge exchange and knowledge sharing by improving people’s understanding and satisfaction of their potential needs. The stronger the reciprocal belief of members of virtual academic community, the more willing they are to participate in knowledge acquisition and exchange activities, and the more willing they are to share more high-quality knowledge with others. Therefore, this paper holds that reciprocity has the following relations with the quantity and quality of knowledge sharing:

• H8: there is a positive correlation between the reciprocity among members of virtual academic community and the quantity of knowledge sharing.
• H9: there is a positive correlation between the reciprocity among members of virtual academic community and the quality of knowledge sharing.

In social organizations, social connection is an important content of social capital structure, and also an important channel for information exchange and knowledge acquisition, representing the strength of a two-way relationship between members. Close social connection means stability, trust, and cooperation, which can promote members’ understanding of the overall objectives and behaviors of the organization, stimulate members’ efforts, and reduce concerns about the effectiveness of knowledge sharing, to ensure the transfer and sharing of high-quality knowledge. Many studies have confirmed the important influence of social contact on information exchange and knowledge sharing. Research on the evaluation of social e-commerce word-of-mouth indicates that social contact can effectively promote user communication, which has a significant impact on the quantity and quality of online word-of-mouth (Goraya et al., 2019). Hall and Merolla (2020) measured social connection from three aspects: communication frequency, time, and closeness, which showed that social connection can stimulate the external motivation of community members and improve the quality of knowledge sharing (Hall and Merolla, 2020). In the virtual academic community, the closely related community members are willing to share more knowledge and higher quality. Accordingly, the following assumptions are proposed:

• H10: there is a positive correlation between the social connection and the quantity of knowledge sharing among members of virtual academic community.
• H11: there is a positive correlation between the social connection among members of virtual academic community and the quality of knowledge sharing.

**FIGURE 1:**
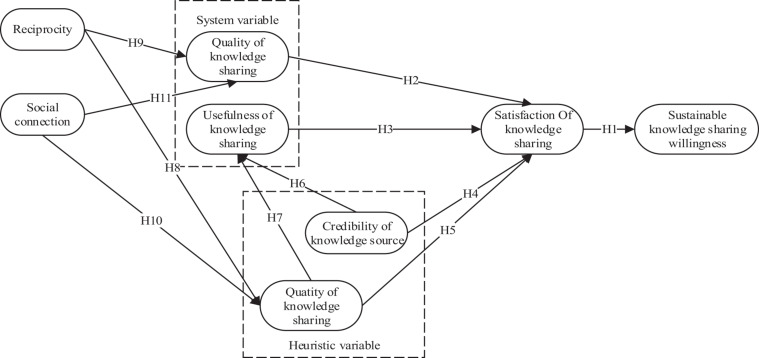
Research model.

Based on the above assumptions, this study proposes the following research model, as shown in Figure 1.

## Materials and Methods

### Selection of Experimental Platform

**FIGURE 2:**
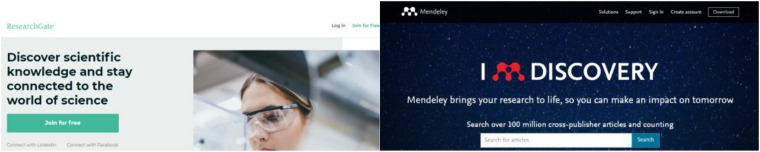
Homepages of ResearchGate and Mendeley.

This study mainly choose ResearchGATE and Mendeley to collect experimental data, see Figure 2. ResearchGate is a professional network composed of scientists and researchers. At present, more than 20 million members from all over the world have used it to share, discover and discuss research. Its main functions are to update research consultation at any time, communicate with researchers in professional fields in time, and provide sustainable learning approaches. At the same time, the platform is free to open research to all people, and has strict privacy protection technology and service aims to ensure the safety of data and shared knowledge. Mendeley is a free reference manager and sharing platform, which has been used by more than one million users. Its main function is to help store, organize, record, share, and quote reference materials and research data. The main advantage of Mendeley is that it can easily collaborate with other researchers online, obtain literature and share opinions from multiple sources. Based on the above introduction of the two platforms, it can be known that both platforms are open to the outside world free of charge and have a large number of users, which is convenient for later sample selection and data collection. At the same time, these two platforms are the international mainstream academic virtual community platforms, which have been recognized by researchers, so it is representative to choose these two platforms.

### Sample Selection

Firstly, during the COVID-19 period, this paper collected 500 demographic information from China (including Taiwan Province and Hong Kong), the United States, Singapore and Indonesia by using the virtual academic community platform. All participants were informed of the purpose of this study and the confidentiality of data at the beginning, and once they filled out the questionnaire of this study, they agreed to participate. Secondly, this study randomly selected 100 people to conduct pre-survey with self-designed scale (see Table A1), its main purpose is to test whether there is any language expression or unclear meaning in the questionnaire. A total of 62 valid questionnaires were collected in the pre-survey, and the researchers adjusted them according to the feedback results of the pre-survey. Finally, because 100 people have been filled out and interviewed in the pre-survey study, in order to ensure the reliability and accuracy of the data, this study excluded these participants in the formal survey, and the remaining 400 people were formally investigated in this study, and 256 valid questionnaires were recovered. In terms of gender, 62% of the respondents were male and 38% were female. In terms of professional titles, professors account for 21%, associate professors account for 32%, and lecturers and graduate students account for 47%; As far as age structure is concerned, 82% are aged 29–45, 8% are over 45, and 10% are under 29. In terms of subject background, the natural sciences accounted for 64%, and the social sciences and humanities accounted for 36%. The study was conducted by the Declaration of Helsinki (2002) and Measures for Ethical Review of Biomedical Research Involving Humans, Ministry of Health, China. The protocol was approved by the Ethics Committee of Nanjing Normal University.

### Procedure

In order to better discuss and measure the knowledge sharing willingness of researchers in virtual academic community, this study conducted a cross-sectional survey of 500 researchers from February 2020 to June 2020. The main experimental design includes four parts: firstly, getting the scale factor structure and research hypothesis according to the results of HSMP and literature review. Secondly, 100 researchers were selected for pre-survey and interview to ensure the accuracy of the scale. Thirdly, the remaining 400 researchers were finally filled out with questionnaires and collected with data. Finally, SPSS and Amos are used to analyze the data and draw a conclusion. It is worth mentioning that the reason why this study adopts online survey is that it is difficult for researchers to collect data face to face due to the outbreak of epidemic.

### Data Processing

In order to determine whether the measurement has satisfactory psychometric attributes, SPSS 25.0 and Amos 24.0 were used to analyze the data. Firstly, descriptive statistics are used to analyze the data distribution and Cronbach α coefficient is used to evaluate the reliability of the scale, so as to judge whether the sample distribution is suitable for the next analysis. Secondly, analyze the correlation among the variables and judge whether the model can be constructed. Finally, the structural equation model is constructed by using Amos 24.0, and the relationship among the variables is discussed.

## Results

### Common Method Deviation Test

In this study, the test scale is used to investigate, and all of them are conducted in a unified way. The content of the questionnaire, the characteristics of the participants and the environment of the test may cause covariation between the efficacy standard and the prediction, which may lead to deviation of the research results. In order to effectively verify the existence of common method deviation, Harman single factor test was adopted in this study, and exploratory factor analysis was made for all items. Through analysis, when the eigenvalue root is greater than 1, the variance explained by the first factor is 17.43% < 40%. Therefore, there is no serious common method de-viation among the variables in this study.

### Reliability and Validity Test of the Measurement Model

The reliability of the measurement model was measured by average variance extracted (AVE), composite reliability (CR), and Cronbach Alpha, with the lowest values of 0.5, 0.7, and 0.7, respectively. As shown in Table 1, the AVE value of all variables is greater than 0.7, the CR of all variables is greater than or equal to 0.886, and the Cronbach’s Alpha of all variables is greater than or equal to 0.777, indicating that the measurement model has good reliability.

**TABLE 1:** Reliability test of the model.

**Variable**	**Numbers**	**AVE**	**CR**	**Cronbach’s Alpha**
Sustainable knowledge sharing willingness	3	0.838	0.941	0.922
Usefulness of knowledge sharing	4	0.798	0.904	0.910
Quality of knowledge sharing	4	0.711	0.886	0.876
Quantity of knowledge sharing	4	0.812	0.921	0.933
Reciprocity	4	0.861	0.957	0.945
Satisfaction Of knowledge sharing	3	0.901	0.961	0.955
Social connection	4	0.891	0.959	0.948
Credibility of knowledge source	4	0.721	0.892	0.777

The validity of the measurement model includes content validity and construct validity. Content validity examines the comprehensiveness and representativeness of the content of the measurement indicators. As the measurement items of all variables come from existing research and are pre investigated in advance, the clarity and relevance of the measurement variables are guaranteed. Construction validity includes aggregation validity and differentiation validity. Aggregation validity is measured by AVE, and the threshold value of AVE is 0.5. According to Table 1, all AVE values are between 0.711 and 0.901, indicating that aggregation validity is good. It can be seen from Table 2 that the square root of the mean-variance of all variables is greater than the correlation coefficient, so the discrimination validity is good.

**TABLE 2:** Correlation coefficient.

**Variable**	**1**	**2**	**3**	**4**	**5**	**6**	**7**	**8**
1. Sustainable knowledge sharing willingness	0.922							
2.Usefulness of knowledge sharing	0.656	0.910						
3.Quality of knowledge sharing	0.533	0.595	0.876					
4.Quantity of knowledge sharing	0.501	0.498	0.622	0.933				
5.Reciprocity	0.612	0.688	0.521	0.567	0.945			
6.Satisfaction of knowledge sharing	0.701	0.599	0.534	0.696	0.589	0.955		
7.Social connection	0.333	0.421	0.383	0.333	0.347	0.466	0.948	
8.Credibility of knowledge source	0.489	0.487	0.524	0.410	0.587	0.481	0.367	0.777

### Fit Analysis of the Structural Model

**FIGURE 3:**
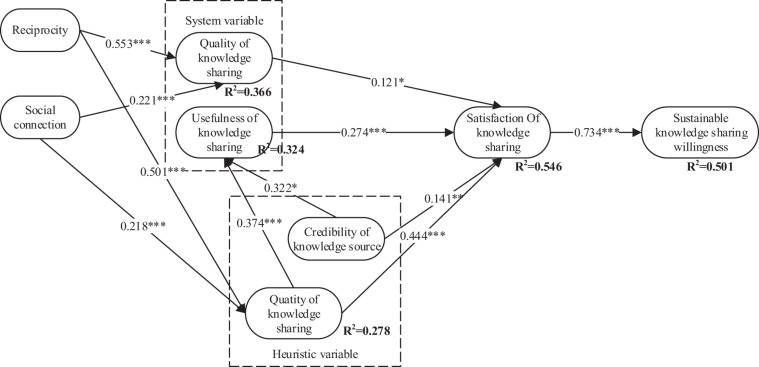
Model results. *p < 0.05, **p < 0.01, ***p < 0.001.

Partial least square method is used to analyze the structural model, including path coefficient among variables, significance degree of the path (all significant paths are marked with ^∗^ mark), and variance of variable interpretation (R2). The analysis results are shown in Figure 3.

The results of the structural model test show that 50.1% of the difference of sustainable willingness of knowledge sharing is caused by the satisfaction of knowledge sharing, and R2 (explained variance) of satisfaction of knowledge sharing is 54.6%, that is to say, 54.6% of the variance of satisfaction of knowledge sharing is explained by various heuristic factors and systematic variables, which shows that the structural model has better prediction effect. Besides, all hypotheses are verified. The satisfaction of knowledge sharing has a significant positive effect on the sustainable willingness of knowledge sharing (β = 0.701, *P* < 0.001). Hypothesis H1 is verified. Systematic factors and heuristic factors are the key predictors of satisfaction of knowledge sharing. Systematic factors include the quality of knowledge sharing and the usefulness of knowledge sharing, and their influence coefficients are 0.121 (*P* < 0.05) and 0.274 (*P* < 0.001), respectively. Heuristic factors include the quantity of knowledge sharing and the credibility of knowledge sources, and their influence coefficients are 0.444 (*P* < 0.001) and 0.141 (*P* < 0.0), respectively 1) (β = 0.444, *P* < 0.001), assuming that H2, H3, H4, H5 are all verified. The quantity of knowledge sharing and the credibility of knowledge source have significant positive effects on the usefulness of knowledge sharing, the influence coefficients are 0.374 (*P* < 0.001) and 0.322 (*P* < 0.001), respectively, assuming that H6 and H7 are tenable. In addition, the two social influence variables of reciprocity and social connection have a significant influence on some heuristic variables (quantity of knowledge sharing) and systematic variables (quality of knowledge sharing), and reciprocity has a significant influence on the quality of knowledge sharing (β = 0.553, *P* < 0.001). And the quantity of knowledge sharing (β = 0.501, *P* < 0.001) had a greater impact. The social connection had a smaller impact on the quality of knowledge sharing (β = 0.221, *P* < 0.001) and the quantity of knowledge sharing (β = 0.218, *P* < 0.001), assuming that H8, H9, H10, H11 were all tenable.

## Discussion

### The HSMP Supports the Related Research of Online Knowledge Sharing in the Future Theory

With the continuous development of COVID-19, it will inevitably lead to an increase in the proportion of online academic exchanges in the future. Due to various discomforts caused by the initial online sharing, the publication of a large number of related studies from 2019 to 2021 can show that researchers attach importance to this issue. However, the existing literature is difficult to provide a reliable theoretical basis to ensure the scientific nature of the research. Therefore, although HSMP is more and more applied to the research of network information behavior, it is less applied in the field of knowledge sharing in virtual academic community, which is an innovation. In this paper, the satisfaction of knowledge sharing and sustainable willingness of knowledge sharing is regarded as a dual process, including heuristic and systematic behaviors, which are affected by heuristic cues (including two heuristic variables of reliability of knowledge source and quantity of knowledge sharing) and systematic cues (including two systematic variables of the usefulness of knowledge sharing and quality of knowledge sharing). Two heuristic variables have a significant impact on the usefulness of knowledge sharing, which is a system variable. The deviation effect of the HSMP is verified. It can be seen that the application of HSMP from the general rules of human behavior decision-making to explore satisfaction of knowledge sharing and sustainable willingness of virtual academic community can reveal the influence and mechanism of various rational and irrational factors on the satisfaction of knowledge sharing and sustainable willingness of virtual academic community. It can also provide a novel and interesting research perspective for more studies on epidemic situation and education in the future.

### Practice Verifies the Influencing Factors of Sustainable Knowledge Sharing

In practice, managers of virtual academic community can make efforts to improve satisfaction of knowledge sharing and sustainable willingness of virtual academic community through four aspects:

#### Quality and Usefulness of Knowledge Sharing

Quality and usefulness of knowledge sharing are two systematic variables of satisfaction evaluation, which have an important impact on satisfaction. For new or unfamiliar virtual communities, users usually adopt systematic behavior mode in the process of knowledge sharing and satisfaction evaluation, mainly based on the quality and usefulness of knowledge sharing for decision-making, which consumes more cognitive ability and resources. Because the virtual academic community is a loose organization formed by self-organization, there is no mandatory constraint mechanism, and there may be intellectual property disputes, timeliness is not strong, innovation is not enough, and the relevance with the discussion topic is not strong in the process of knowledge sharing. At the same time, the amount of community knowledge is large and growing constantly, and community members are inconvenient to obtain high-quality knowledge, thus reducing the usefulness of knowledge sharing. In view of this, the virtual academic community can identify the potential high-quality content by using the combination of machine algorithm and artificial screening, use knowledge mining and semantic retrieval technology to achieve the rapid acquisition of community knowledge, and carry out semantic analysis and deep-seated aggregation of community knowledge, build a knowledge navigation system with interrelated content, multi-dimensional and multi-level, and provide deep-seated knowledge services To improve the effectiveness and usefulness of knowledge sharing.

#### The Credibility of Knowledge Source and Quantity of Knowledge Sharing

The credibility of knowledge source and quantity of knowledge sharing are two important heuristic variables. For virtual communities with a certain degree of social recognition, in the process of satisfaction evaluation and sustainable willingness formation of user knowledge sharing, community members usually follow the principle of minimum effort and tend to adopt heuristic behavior mode, mainly based on the source (credibility) and surface characteristics (quantity of knowledge sharing) It costs less cognitive effort and resources to judge. Therefore, the managers of virtual academic community can reduce the cognitive burden of users by increasing the credibility of knowledge sources and the number of knowledge: first, adopt the real-name system to improve the credibility of users and build a high-quality community; second, use PageRank, hits and other link algorithms for reference, comprehensively consider the academic authority and community influence of users, and calculate the ranking value of community user credibility (Person Rank, PR); the third is to meet the user’s human needs to share knowledge and build prestige as much as possible, learn from the experience of community, improve the possibility of new users being recognized, and encourage users to continue to participate in knowledge sharing activities.

#### The Satisfaction of Knowledge Sharing and Sustainable Willingness

Heuristic behavior and systematic behavior can occur at the same time. The process of satisfaction of knowledge sharing evaluation and sustainable willingness formation has the characteristics of both heuristic and systematic behavior patterns, making the results both intuitive and rational. Community members determine behavior patterns mainly according to the motivation and ability factors in specific situations and seek the relative optimal solution in the process of weighing the minimum cognitive effort and the maximum benefit. In this regard, managers of virtual academic community need to consider the balance between knowledge sharing benefits and cognitive costs, pay attention to collecting and saving knowledge sharing behavior tracks and relevant data, and use big data technology and methods to deeply mine the professional characteristics, research preferences and behavior habits of community members, so as to provide an intelligent recommendation of knowledge sharing, so that community members can have the minimum cognitive cost get the most from knowledge sharing.

#### Reciprocity and Social Connection

Heuristic cues and systematic cues are affected by external social capital. Reciprocity and social connection have a significant influence on the quantity and quality of knowledge sharing, and the influence of reciprocity is greater than that of social connection. Knowledge sharing in virtual academic community is a collective exchange behavior among members, and the pursuit of interests is the key factor to promote the exchange behavior. The interests here include not only material rewards, but also psychological rewards such as self-esteem, approval, support, and prestige, and psychological rewards are usually more important than material rewards. Because of this, the managers of virtual academic community need to take effective measures. For example, establish a weak relationship based on interest, hold offline activities, promote mutual communication and recognition, improve trust among members, enhance social contact and community activity among members, and improve the knowledge sharing effect of virtual academic community.

### Knowledge Sharing Under Epidemic Situation Helps Online Academic Development

With the outbreak of the epidemic, more and more online tools have been developed. The main goal is to allow users to create and participate in academic activities through communication, sharing, collaboration, publishing, management and interaction. Among these key functions, sharing has always been regarded as an important component of social media, and the sustainable sharing will affect its future development trend. As one of the mainstream social media tools for academic communication, the sharing of knowledge and information has become one of its basic functions. Knowledge sharing is defined as the process of individuals spreading knowledge to others, which essentially shows that knowledge sharing needs social interaction. However, knowledge sharing involves the behavior that individuals make others have their own proprietary technology and information sources, so it is very important to promote personal willingness to share knowledge.

The results of this study show that researchers are optimistic about knowledge sharing in virtual academic communities. Online knowledge sharing makes it easy for researchers to obtain cutting-edge knowledge and encourage each other from other researchers, while cutting-edge knowledge and friendly interpersonal relationships can enable researchers to actively consider the value of knowledge sharing as an academic activity, and also help them to conduct academic research better under many difficulties caused by the epidemic. However, this social effect depends on whether researchers regard the platform as a shared platform, because different individuals may perceive the same technology differently, which may subsequently affect the way they interact with the technology. Therefore, the extent to which researchers think that the platform provides easy online knowledge sharing may also determine the possibility or even the sustainability of their willingness to regard the platform as a valuable academic tool. This is also a meaningful focus for further investigation in future research.

### How to Effectively Build a Virtual Academic Community and Help Learning

With the outbreak of the epidemic, online learning platform and online effective learning have been widely concerned. For a wide range of academic researchers, a complete and effective academic community platform has become an indispensable tool for future research. How to create a complete academic community platform and improve the use effect of online learning should be discussed from three aspects: researchers’ sustainable willingness to participate, academic community managers’ attention and input support, and the quality of online learning products.

Firstly, the results of this study show that researchers’ sustainable willingness to share knowledge is at a high level, which reflects that the reason why a platform is used for a long time is influenced by researchers’ willingness to use it. Therefore, in the development of academic community, it is necessary to pay close attention to users’ use feelings and problem feedback at any time, and timely handle and solve problems to ensure that researchers’ use feelings will not be greatly affected.

Secondly, the orientation and function of academic community need to keep pace with the times to ensure the forefront, which is consistent with the needs of researchers. Therefore, it involves the management and maintenance of academic communities by managers, who must ensure the smooth use of platform functions, update and expand the resources needed by researchers in a timely manner, and strictly control the protection of research data and scientific research achievements. That’s why this research chooses ResearcheGate and Mendeley as research platforms, because they do well enough to ensure that there are millions of users.

Finally, if individuals want to ensure effective online learning, they should be clear about why they learn. Online learning requires a higher level of self-control. Therefore, researchers should make a complete study plan before studying. In the process of learning, the academic community can provide researchers with professionals in the same professional field, from which you can discuss and share the confusion and experience of learning, which will help you deepen understanding of the content and maintain continuous enthusiasm for learning. Mendeley, for example, can comment on the literature read online and share it with the study group in time. In addition to studying, it is difficult for us to communicate face to face due to the epidemic situation, and the online virtual academic community provides us with the function of online meeting. Therefore, researchers should keep an optimistic attitude toward learning and a correct willingness to share in order to ensure that everyone can obtain accurate information.

## Limitations

There are some limitations in explaining the current study. First, during the period of COVID-19, data were available only through online tools. Although the scale of this study is submitted to the virtual academic community users to fill in and retrieve in time, there may be some deviation in the data basis. Secondly, the sample size of this study is limited. Perhaps a larger sample size will make this study more effective. Finally, although the results of this study confirm the relationship between sustainable share knowledge willingness and some variables, is this result more serious during the outbreak than before? Since this study cannot obtain pre epidemic data, it is impossible to compare and analyze the willingness to share knowledge before and after the outbreak, but this study can explore the future data after the epidemic situation is stable. Therefore, the future research focus of this paper will also explore whether the sustainable knowledge sharing willingness of virtual academic community will be different from that during the epidemic and whether there is a more direct relationship with other factors when the epidemic is over, offline academic exchanges and knowledge sharing activities are fully restored.

## Data Availability Statement

The original contributions presented in the study are included in the article/supplementary material, further inquiries can be directed to the corresponding author/s.

## Ethics Statement

The studies involving human participants were reviewed and approved by the Ethics Committee of Nanjing Normal University. The patients/participants provided their written informed consent to participate in this study.

## Author Contributions

All authors listed have made a substantial, direct and intellectual contribution to the work, and approved it for publication.

## Conflict of Interest

The authors declare that the research was conducted in the absence of any commercial or financial relationships that could be construed as a potential conflict of interest.

## Appendix

**TABLE TA1:** Scale of sustainable knowledge sharing in virtual academic community.

**Serial number**	**Topic**
1	I like to help others by sharing my knowledge.
2	I am confident that I can provide valuable knowledge to others.
3	I have the ability to provide valuable knowledge.
4	I believe that a solid knowledge base is easier to accept new knowledge.
5	I am good at sharing new knowledge to work or study.
6	I am good at applying new methods of knowledge sharing to work or study.
7	Shared knowledge is helpful to my work or study.
8	Shared knowledge is related to the topic.
9	Shared knowledge is rigorous and accurate.
10	Shared knowledge is complete.
11	Shared knowledge is timely.
12	Rich knowledge topics provided by virtual community.
13	Sufficient knowledge provided by virtual community.
14	The knowledge shared by virtual community contains abundant information.
15	The knowledge topic of virtual community updating and sharing.
16	I am willing to share knowledge.
17	I am not willing to share knowledge.
18	I would like to participate in the discussion of virtual community.
19	I would like to respond to the topic.
20	I am satisfied with virtual community products.
21	I am satisfied with virtual community service.
22	I am satisfied with the use of virtual communities.
23	I can find the same professional in the virtual community.
24	I have people in close contact with the virtual community.
25	I can build friendships in virtual communities.
26	I belong to a topic organization of virtual community.
27	The knowledge shared by virtual community is based on my willingness.
28	The knowledge shared by virtual community is authoritative.
29	Knowledge recognition of virtual community sharing is high.
30	The knowledge shared by virtual community should be trusted.
